# Experimental and Computational Analysis of Phenolic Acid Association with PAMAM Dendrimers: Comparing Different Formulation Techniques

**DOI:** 10.3390/polym18091086

**Published:** 2026-04-29

**Authors:** Christopher Sbarbaro, Ma. Andreina Rangel-Ramírez, Emilio Salas, Francisco Salgado, María Carolina Otero, Alvaro A. Elorza, Fernando González-Nilo, Valeria Márquez-Miranda, Yorley Duarte

**Affiliations:** 1 Center of Bioinformatics and Integrative Biology, Facultad de Ciencias de la Vida, Universidad Andres Bello, Santiago 8370035, Chile; christopher.sbarbaro@usach.cl (C.S.); e.salasaguilar@uandresbello.edu (E.S.); fsalgado@uchile.cl (F.S.); fernando.gonzalez@unab.cl (F.G.-N.); 2 Departamento de Ciencias Biológicas, Facultad de Ciencias de la Vida, Universidad Andrés Bello, Santiago 8370035, Chile; rangelmariandre@gmail.com; 3 Escuela de Química y Farmacia, Facultad de Medicina, Universidad Andrés Bello, Santiago 8370035, Chile; maria.otero@unab.cl; 4 Instituto de Ciencias Biomédicas, Facultad de Medicina y Ciencias de la Vida, Universidad Andrés Bello, Santiago 8370035, Chile; alvaro.elorza@unab.cl; 5 Instituto de Neurociencias, Centro Interdisciplinario de Neurociencias de Valparaíso, Universidad de Valparaíso, Valparaíso 2360102, Chile

**Keywords:** encapsulation, nanomedicine, computational modeling, nanocarrier, drug delivery, antioxidant activity, phenolic compounds

## Abstract

Poly(amidoamine) (PAMAM) dendrimers are widely recognized as versatile nanocarriers due to their tunable architecture and ability to associate with bioactive molecules. In this study, generation 3 PAMAM dendrimers functionalized with triphenylphosphonium (TPP) were employed to investigate the association of structurally related phenolic compounds—caffeic acid, p-coumaric acid, and cinnamic acid—through either covalent conjugation or non-covalent encapsulation. Physicochemical characterization by NMR, dynamic light scattering, and zeta potential measurements revealed the formation of supramolecular aggregates rather than isolated dendrimer units, with hydrodynamic diameters ranging from 127 to 260 nm and positive surface charge across all formulations. Encapsulation efficiencies determined by HPLC reached 93.8% for caffeic acid, 78.9% for p-coumaric acid, and 71% for cinnamic acid, indicating differential association behavior. Molecular dynamics simulations over 1 μs supported these findings, showing stronger and more stable interactions for polar antioxidants, particularly caffeic acid, driven by hydrogen bonding and electrostatic interactions, while cinnamic acid displayed preferential binding in more hydrophobic dendrimer regions. Radical scavenging assays (DPPH• and ABTS•+) demonstrated that all formulations retained antioxidant capacity, although dendrimer association modulated scavenging kinetics. In cellular assays under oxidative stress, free caffeic acid exhibited the strongest immediate reduction of intracellular reactive oxygen species, whereas dendrimer-associated systems showed reduced but significant activity, consistent with decreased solvent accessibility and slower release predicted by simulations. Overall, these results highlight a trade-off between molecular retention and immediate biological efficacy, demonstrating that the mode of association governs antioxidant accessibility and performance. This combined experimental and computational approach provides a mechanistic framework for the rational design of dendrimer-based delivery systems aimed at balancing stability and functional activity.

## 1. Introduction

**Figure 1:**
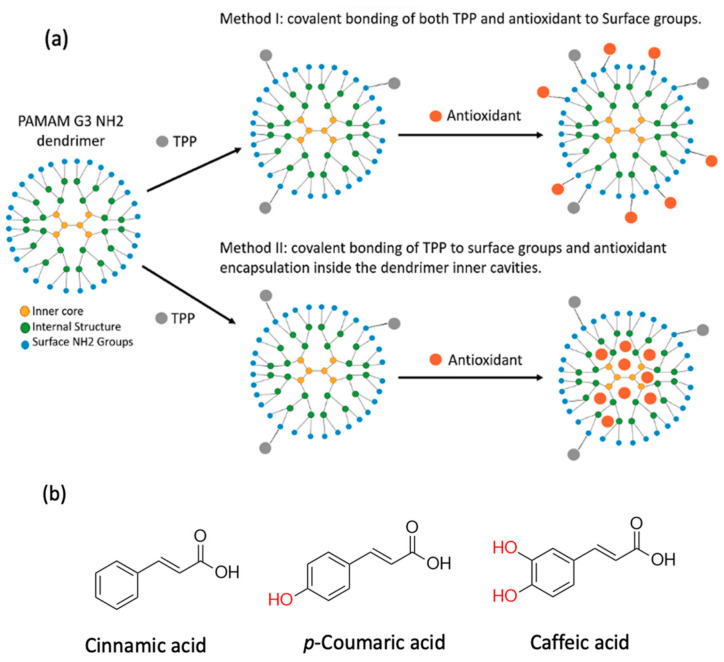
(**a**) Schematic representation of the two strategies used to prepare PAMAM-Antioxidant formulations. Method I represent a covalent conjugation of both TPP and the antioxidant to the surface of NH_2_ groups, while method II showcases covalent conjugation of TPP to the dendrimer surface groups, followed by a non-covalent encapsulation of the antioxidant molecules inside the internal cavities of PAMAM. (**b**) Chemical structures of phenolic antioxidants used in this study, together with a structurally related non-phenolic analogue, displaying their differing number of phenolic hydroxyl groups.

In recent years, dendrimers have emerged as promising platforms for the association and stabilization of bioactive compounds, owing to their structural and chemical properties, such as a well-defined size, tunable surfaces for specialized functionalization, and monodispersity in solution [1,2]. In particular, polyamidoamine (PAMAM) polymers have demonstrated high biocompatibility, favorable solubility, and the capacity to encapsulate hydrophobic drugs or therapeutic agent conjugates that otherwise struggle to exert effects due to their unfavorable physicochemical properties [3]. Internal cavities present in PAMAM, along with their spherical morphology with size comparable to those of other macromolecules such as proteins (e.g., G_3_–G_5_ dendrimers mimic insulin to hemoglobin in size [4]), have the potential to enhance biocompatibility and reduce immunogenicity. For these reasons, PAMAM dendrimers are an attractive solution for targeted drug delivery (Figure 1), as demonstrated in cancer therapy, where ligand-functionalized systems have improved tumor-specific accumulation [5,6,7].

Mitochondria have been widely investigated as targets for antioxidant delivery due to their central role in cellular energy metabolism and reactive oxygen species (ROS) production, processes implicated in several pathological conditions such as neurodegenerative diseases, cancer, and age-related macular degeneration (AMD) [8,9]. Delivering bioactive compounds to this organelle remains challenging because of the mitochondrial double-membrane structure and the large electrochemical potential across the inner membrane. Among the strategies explored in this context, triphenylphosphonium (TPP) derivatives have been frequently employed due to their lipophilic cationic character, which allows accumulation driven by the mitochondrial membrane potential (−150 to −180 mV) [10,11]. TPP-functionalized probes and carriers have therefore been widely investigated in different biological systems, illustrating the versatility of this structural motif [12]. In this work, TPP was incorporated into the PAMAM dendrimer surface as a representative functional group commonly used in dendrimer-based delivery systems, with a focus on physicochemical characterization.

Despite their promising physicochemical properties, the clinical translation of cationic PAMAM dendrimers is constrained by their inherent cytotoxicity, which arises primarily from electrostatic interactions between the protonated surface amines and the anionic phospholipid headgroups of cellular membranes. These interactions can induce nanoscale membrane defects, bilayer thinning, and increased membrane permeability, ultimately compromising cell viability at therapeutically relevant concentrations. Surface modification strategies—such as PEGylation, acetylation, or conjugation of targeting moieties—are widely investigated as means to attenuate this toxicity while preserving desirable encapsulation and targeting properties. In the present work, partial functionalization of the PAMAM surface with TPP groups and phenolic antioxidant molecules reduces the density of free cationic amines available for membrane interaction.

Among the wide range of bioactive compounds presenting the capacity to scavenge ROS, phenolic acids are highlighted as potent antioxidants, but face challenges related to their bioavailability and stability, granting opportunity for nanocarriers to design novel systems for delivery [13,14]. For instance, gallic acid encapsulation in PAMAM dendrimers (G_4_/G_5_) improved efficiency in radical scavenging, as shown by DPPH• (IC_50_ 0 21.0 ± 4.0 μM vs. 41.7 ± 18 μM of free gallic acid) and ABTS•+ assays, while also reducing cytotoxicity in retinal cells [15]. Similarly, PAMAM-based conjugates with caffeic acid have been able to carry a more efficient wound closure and healing activity by promoting cell proliferation when exposed to HaCaT cells [16]. Systems of co-delivery based in PAMAM have also been explored; for example, PAMAM co-loaded with doxorubicin and siRNA showed synergistic antitumor effects [17]. Although these studies highlight the versatility of PAMAM dendrimers as molecular carriers, prior reports evaluate either covalent conjugation or non-covalent encapsulation independently, frequently focusing on a single compound or formulation strategy. Thus, there remains a limited mechanistic understanding of how the mode of association between dendrimer and guest molecule influences supramolecular organization and functional antioxidant performance. Furthermore, relatively few studies integrate experimental characterization with molecular dynamics simulations to directly relate dendrimer–guest interactions to biological outcomes.

In parallel with experimental approaches, computational modeling has emerged as a key tool to understand and predict the supramolecular behavior of dendrimer–drug systems [18,19]. Molecular dynamics (MD) simulations enable the exploration of atomistic-level interactions that govern encapsulation, diffusion, and conformational stability [20]. Previous studies have demonstrated that PAMAM dendrimers exhibit dynamic cavities whose polarity and volume fluctuate according to solvent environment and guest molecule properties, influencing encapsulation efficiency and release kinetics [21,22]. Furthermore, binding free energy calculations based on MM/PBSA or MM/GBSA approaches have revealed that hydrogen bonding, π–π stacking, and electrostatic interactions are dominant contributors to complex stability in PAMAM–phenolic systems [23,24]. These computational insights complement experimental observations and support a rational design of nanocarriers with optimized affinity and release behavior.

In the present study, caffeic acid and *p*-coumaric acid were selected as representative phenolic antioxidants with differing degrees of hydroxylation, allowing a structure–activity comparison of how substitution pattern influences dendrimer interaction and antioxidant behavior. Cinnamic acid, a structurally related analogue lacking phenolic hydroxyl groups, was intentionally included as a non-phenolic structural analogue to probe the contribution of hydroxylation to binding, encapsulation stability, and functional activity. Both covalent conjugation and non-covalent encapsulation were explored as complementary formulation strategies, as conjugation is expected to enhance molecular retention while potentially restricting accessibility of reactive groups, whereas encapsulation may preserve molecular flexibility but reduce stability. A direct comparison of these two strategies within the same dendrimer platform therefore provides a rational framework to understand how molecular organization governs functional antioxidant performance. In this work, we present a combined experimental and computational study comparing covalent conjugation and non-covalent encapsulation of structurally related compounds within TPP-functionalized generation 3 PAMAM dendrimers. By integrating physicochemical characterization, radical-scavenging assays, cellular evaluation, and molecular dynamics simulations, we aim to establish a mechanistic link between dendrimer–guest interaction and organization.

## 2. Materials and Methods

### 2.1. Materials

Generation 3 poly(amidoamine) (PAMAM) dendrimer (ethylenediamine core), (4-carboxybutyl)triphenylphosphonium bromide (TPP), 3-dimethylaminopropyl-N’-ethylcarbodiimide hydrochloride (EDC·HCl), *N*-hydroxysuccinimide (NHS), caffeic acid, *p*-coumaric acid, cinnamic acid, fluorescein isothiocyanate (FITC), and *p*-toluenesulfonic acid (*p*-TSA) were purchased from Sigma-Aldrich (St. Louis, MO, USA), all of analytical grade. Solvents dimethyl sulfoxide (DMSO, analytical grade) and methanol (HPLC grade) were obtained from Los Alquimistas (Santiago, Chile). Dialysis membranes (SnakeSkin™, 3.5 kDa MWCO) and Amicon Ultra-15 centrifugal filters (3 kDa MWCO) were purchased from Merck (Darmstadt, Germany). 2,2-diphenyl-1-picryhydrazyl (DPPH•) and 2,2′-azino-bis(3-ethylbenzothiazoline-6-sulfonic acid) (ABTS) for radical scavenging activity were obtained from Sigma-Aldrich as well. Cell culture media, reagents (DMEM, FBS, PBS, MTT) and cell lines (HeLa, HEK-293, and COS-7) were acquired from Merck and maintained under standard culture conditions. Computational analyses employed NAMD 3.0 for molecular dynamics simulations, Gaussian 16 for quantum–mechanical parameterization, and VMD 1.9.4 with the Force Field Toolkit v1.3 for CHARMM parameter generation and validation.

### 2.2. Conjugation of TPP to Generation 3 PAMAM-NH_2_ Dendrimer

**Figure 2:**
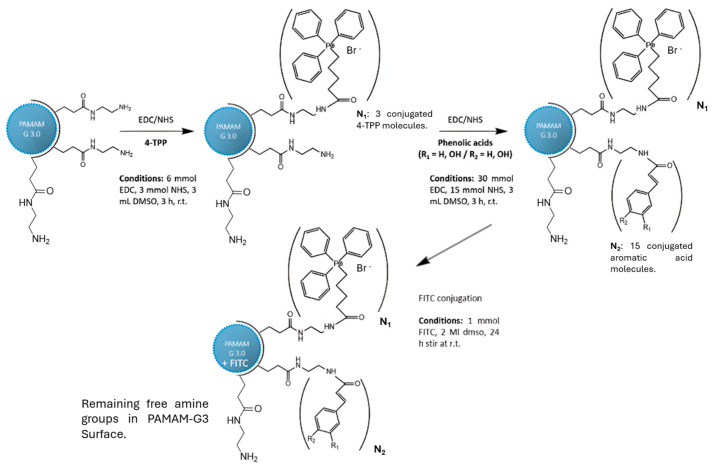
Sequential conjugation scheme used to obtain PAMAM-TPP-antioxidant complexes, employing EDC/NHS-mediated amide bond formation. Step 1 shows conjugation of ~3 TPP groups to surface NH_2_ groups of G3 PAMAM (32 NH_2_ total), leaving approximately unreacted surface primary amine groups. Step 2 illustrates covalent attachment of antioxidants to remaining NH_2_ groups (Method I) or non-covalent encapsulation within the dendrimer interior (Method II). Bromide (Br^−^) is the counterion associated with the TPP quaternary phosphonium group.

The conjugation of TPP to PAMAM-NH_2_ was carried out by EDC/NHS-mediated amide coupling, as depicted in Figure 2 [25]. Three equivalents of (4-carboxybutyl)triphenylphosphonium bromide (TPP), 3 equivalents of EDC·HCl, and 3 equivalents of NHS were dissolved in 3 mL of DMSO and stirred under inert nitrogen atmosphere for 3 h to form the NHS-ester intermediate. In a separate flask, generation 3 PAMAM-NH_2_ (250 µL, 20% *w/v* in methanol) was evaporated at 40 °C for 30 min and redissolved in 3 mL DMSO. The PAMAM solution was added dropwise to the activated TPP mixture and stirred continuously for 72 h at room temperature under nitrogen. The crude product was purified by dialysis against deionized water (SnakeSkin™ membrane, 3.5 kDa MWCO) for 72 h with water changes every 12 h to ensure complete removal of unreacted TPP, EDC, NHS, and urea by-products. The dialyzed solution was lyophilized to yield a white solid (PAMAM-TPP). The degree of TPP substitution was estimated from ^1^H NMR integration by comparing the aromatic proton signals of TPP (δ 7.6–7.9 ppm, phenyl rings) with the methylene backbone signals of PAMAM (δ 2.2–3.5 ppm), yielding an average of approximately 3 TPP groups per dendrimer molecule. The lyophilized product was dissolved in methanol and stored at −20 °C until further use.

### 2.3. Covalent Conjugation of Antioxidants to PAMAM-NH_2_-TPP Structure

The covalent conjugation of caffeic acid, p-coumaric acid, and cinnamic acid to PAMAM-TPP was performed using EDC-mediated amide coupling analogous to that described in Section 2.2. Briefly, 1 equivalent of each antioxidant was activated in 3 mL DMSO by addition of 3 equivalents of EDC and 3 equivalents of NHS, and stirred under inert nitrogen atmosphere for 3 h to form the corresponding NHS-ester intermediates. In parallel, 1 equivalent of PAMAM-TPP was dissolved in 3 mL DMSO and stirred under inert conditions. The activated antioxidant solution was added dropwise and stirred continuously at ambient temperature for 72 h. The resulting conjugates were dialyzed against deionized water for 72 h (3.5 kDa MWCO, SnakeSkin™, Merck) with water changes every 12 h. Dialyzed samples were lyophilized to yield the final solid conjugates, designated Caf/PAM(c), Cou/PAM(c), and Cin/PAM(c). The degree of antioxidant conjugation was estimated from ^1^H NMR by comparing the integration of aromatic/vinylic signals (δ 6.1–7.8 ppm, characteristic of the cinnamate scaffold) relative to the PAMAM backbone signals (δ 2.2–3.5 ppm). This analysis indicated the incorporation of approximately 1–2 antioxidant molecules per dendrimer on average, consistent with the 1:1 molar ratio employed and the partial occupancy of remaining surface NH_2_ groups. Given that ~29 NH_2_ groups remain after TPP conjugation, covalent antioxidant attachment under these stoichiometric conditions is expected to be sub-stoichiometric, leaving the majority of surface amines free and protonatable (see also Figure 2 and Section 3.1).

### 2.4. Encapsulation of Antioxidants Within PAMAM-NH_2_-TPP Structure

Antioxidants caffeic acid (Caf), cinnamic acid (Cin) and *p*-coumaric acid (Cou) were incorporated into the reaction mixture using the molar proportion 1:15 PAMAM:antioxidant in three separate batches. Caf, Cin, and Cou were dissolved separately in 2 mL of DMSO and then slowly added under inert conditions to each PAMAM-TPP batch. The reaction was stirred continuously for 72 h. The resulting product was then filtered through Amicon Ultra-15 centrifugal filters (MWCO 3000 Da, Merck Millipore Ltd., Burlington, MA, USA) against milli-Q grade purified water. The product was frozen overnight and lyophilized to obtain the solid products, designated as Caf/PAM(e), Cou/PAM(e), and Cin/PAM(e). PAMAM formulations were characterized by ^1^H-NMR (Varian Mercury 400 mHz, Palo Alto, CA, USA). Light Scattering (LS) was performed using Malvern Zetasizer (Malvern Panalytical Ltd., Malvern, UK) to determine the product’s molecular diameter and zeta potential. ^1^H-NMR was conducted with 10 mg dissolved in d-DMSO. 1 mg/mL of PAMAM-TPP-Antioxidant was dissolved in deionized water for LS analysis. The PAMAM-TPP-Antioxidant complex was then used as a reactant in the addition of FITC, while the unused PAMAM-TPP-Antioxidant product was left in DMSO and stored at −20 °C until needed.

### 2.5. Entrapment Efficiency of Antioxidant Encapsulates

Entrapment efficiency of PAMAM complexes was performed for formulations prepared using the encapsulation method, calculating it through the amount of unencapsulated antioxidant remaining in the supernatant after filtration. Briefly, the reaction mixtures obtained after encapsulation (Section 2.4) were centrifuged and filtered using Amicon Ultra-15 centrifugal filters (3 kDa MWCO, Merck) at 400× *g* for 10 min. The filtrates containing free (non-entrapped) antioxidants were analyzed by high-performance liquid chromatography (HPLC) using an Agilent 1260 Infinity II HPLC system (Agilent Technologies, Santa Clara, CA, USA) equipped with a diode array detector (DAD). Separations were carried out on a SunShell C18 column (150 mm × 4.6 mm i.d., 2.6 μm particle size) at 30 °C, under isocratic conditions. The mobile phase consisted of methanol: water (70/30%*v*/*v*), delivered at a flow rate of 1.0 mL/min. Detection wavelengths were set to 325 nm for caffeic acid, 310 nm for *p*-coumaric acid, and 295 nm for cinnamic acid. Calibration curves were established for each antioxidant using standard solutions with concentrations between 1 and 60 μM, yielding equations. Entrapment efficiencies were calculated by comparing the amount of antioxidant present in the supernatant (unentrapped fraction) with the total amount initially used prior to encapsulation. The difference between these two values represented the quantity of encapsulated compound, and efficiency was expressed as a percentage of the initial concentration. The results were compared using prepared calibration curves for each standard, prepared in a range of concentrations between 1 and 60 µM (fitting equations for caffeic, coumaric, and cinnamic acid standards where y = 26,336x − 380.6, y = 73.96x − 4.081 and y = 10,154x − 2001, with R2 values of 0.9960, 0.9967 and 0.9951, respectively).

### 2.6. Structural Characterization by NMR

NMR Spectroscopy was used to reveal shifts that correspond to both the corresponding antioxidants and the presence of the main PAMAM structure. For ^1^H-NMR and ^13^C-NMR measurements, each sample was dissolved in deuterated methanol (MeOD) at the desired concentration and transferred to 5 mm NMR tubes prior to analysis. The lower TPP proportion and presence in relation to the other two components can be confirmed through the shifts observed at 1.5–2 ppm, which are characteristic of the hydrocarbon chain of the TPP structure. ^13^C-NMR data is also displayed [26]. Due to the highly branched architecture of PAMAM dendrimers, the methylene groups of the dendrimer backbone generate broad and partially overlapping signals in the aliphatic region of the ^1^H NMR spectra. These resonances are typically observed between δ 2.2–3.5 ppm and correspond to the tertiary and secondary amine-linked methylene units of the PAMAM framework. In contrast, signals appearing in the aromatic region between δ 6.5–7.8 ppm correspond to the phenolic acid moieties incorporated into the dendrimer systems. In particular, the presence of vinylic doublets with coupling constants around J ≈ 15–16 Hz is consistent with the trans-olefinic protons of the cinnamic, caffeic, and p-coumaric acid derivatives. The residual solvent signal corresponding to MeOD (δ ≈ 3.31 ppm) was identified.

PAMAM-Caffeic Acid: ^1^H NMR (400 MHz, MeOD) δ 8.00 (s, ^1^H), 7.56–7.34 (m, ^1^H), 7.05 (d, J = 2.0 Hz, ^1^H), 6.93 (dd, J = 8.3, 2.1 Hz, ^1^H), 6.79 (d, J = 8.1 Hz, 2H), 6.36–6.19 (m, ^1^H), 3.33 (d, J = 1.7 Hz, 27H), 2.83 (s, 16H), 2.68 (s, 47H), 2.62 (d, J = 6.2 Hz, 2H), 2.41 (s, 17H). ^13^C NMR (101 MHz, MeOD) δ 172.8, 134.9, 133.5, 133.4, 130.2, 130.1, 115.2, 52.1, 50.9, 49.8, 47.6, 47.4, 47.2, 46.9, 39.1, 38.6, 36.3, 34.4.

PAMAM-Coumaric Acid: ^1^H NMR (400 MHz, MeOD) δ 7.41–7.37 (m, ^1^H), 7.34 (s, ^1^H), 6.84–6.76 (m, ^1^H), 6.35 (d, J = 15.9 Hz, ^1^H), 3.33–3.22 (m, 2H), 2.94 (d, J = 5.9 Hz, 2H), 2.79 (t, J = 6.8 Hz, 4H), 2.61–2.54 (m, 2H), 2.38 (q, J = 7.8 Hz, 4H). ^13^C NMR (101 MHz, MeOD) δ 140.7, 128.9, 115.3, 52.0, 49.7, 38.8, 37.2, 33.3. 13C NMR (101 MHz, MeOD) δ 140.74, 128.95, 115.31, 52.03, 49.72, 38.83, 37.25, 33.33.

PAMAM-Cinnamic Acid: ^1^H NMR (400 MHz, MeOD) δ 7.57–7.49 (m, ^1^H), 7.38–7.35 (m, ^1^H), 7.35–7.30 (m, ^1^H), 6.53 (d, J = 15.9 Hz, ^1^H), 3.33–3.23 (m, 3H), 2.95 (t, J = 6.0 Hz, 2H), 2.81 (t, J = 6.8 Hz, 5H), 2.63–2.55 (m, 3H), 2.40 (q, J = 5.7 Hz, 5H). ^13^C NMR (101 MHz, MeOD) δ 172.8, 159.5, 140.4, 134.8, 128.6, 127.5, 50.8, 49.7, 38.9 (d, J = 37.3 Hz), 37.2, 33.2.

PAMAM-TPP-Caffeic Acid (covalent): ^1^H NMR (400 MHz, MeOD) δ 7.74–7.60 (m, ^1^H), 7.39 (d, J = 15.9 Hz, ^1^H), 6.94 (d, J = 2.1 Hz, ^1^H), 6.88–6.78 (m, ^1^H), 6.68 (d, J = 8.2 Hz, ^1^H), 6.13 (d, J = 15.9 Hz, ^1^H), 3.25 (s, ^1^H), 2.94 (s, 2H), 2.77 (s, ^1^H), 2.48–2.37 (m, ^1^H), 1.84–1.70 (m, ^1^H).

PAMAM-TP*p*-coumaric Acid (covalent): ^1^H NMR (400 MHz, MeOD) δ 7.67 (d, J = 16.4 Hz, 4H), 7.31–7.22 (m, ^1^H), 6.68 (t, J = 9.1 Hz, ^1^H), 6.22 (d, J = 16.0 Hz, ^1^H), 3.58–3.50 (m, ^1^H), 3.30 (s, 4H), 3.00–2.90 (m, ^1^H), 2.67 (s, 8H), 2.47 (s, 3H), 2.26 (s, 9H), 1.73 (s, ^1^H), 1.58 (s, ^1^H). ^13^C NMR (101 MHz, MeOD) δ 140.5, 134.9, 133.4, 133.3, 130.2, 130.1, 115.4 (d, J = 25.8 Hz), 52.0, 49.7, 39.0, 37.2, 33.3.

PAMAM-TPP-Cinnamic Acid (covalent): ^1^H NMR (400 MHz, MeOD) δ 7.68 (d, J = 15.9 Hz, ^1^H), 7.45–7.37 (m, ^1^H), 7.35–7.19 (m, 2H), 6.41 (d, J = 16.0 Hz, ^1^H), 3.58–3.40 (m, ^1^H), 3.33 (s, 2H), 2.93 (s, ^1^H), 2.68 (s, 4H), 2.47 (s, 2H), 2.27 (s, 5H), 1.73 (s, ^1^H), 1.58 (s, ^1^H). ^13^C NMR (101 MHz, MeOD) δ 140.3, 133.4 (d, J = 10.1 Hz), 130.2, 130.1, 128.6, 128.4, 127.5, 127.1, 52.0, 49.7, 39.0, 37.2, 33.3.

PAMAM-TPP-Caffeic Acid (encapsulated): ^1^H NMR (400 MHz, MeOD) δ 7.37 (d, J = 15.8 Hz, ^1^H), 6.93 (d, J = 2.2 Hz, ^1^H), 6.82 (d, J = 2.1 Hz, ^1^H), 6.80 (d, J = 2.1 Hz, ^1^H), 6.67 (d, J = 8.2 Hz, ^1^H), 6.14 (d, J = 15.9 Hz, ^1^H), 3.49 (dd, J = 11.4, 4.6 Hz, ^1^H), 3.41–3.35 (m, ^1^H), 3.19 (s, 2H), 2.70 (s, 2H), 2.49 (d, J = 15.0 Hz, ^1^H), 2.28 (s, 2H), 1.77 (dt, J = 13.8, 6.9 Hz, ^1^H), 1.54 (s, ^1^H). ^13^C NMR (101 MHz, MeOD) δ 147.7, 145.3, 144.3, 126.7, 121.1, 115.9, 115.1, 113.6.

PAMAM-TP*p*-coumaric Acid (encapsulated): ^1^H NMR (400 MHz, MeOD) δ 7.48 (d, J = 15.9 Hz, ^1^H), 7.45–7.38 (m, 2H), 6.86–6.77 (m, 2H), 6.33 (d, J = 15.9 Hz, ^1^H), 3.68–3.55 (m, ^1^H), 3.31 (d, J = 11.5 Hz, 5H), 3.12 (d, J = 6.1 Hz, ^1^H), 2.81 (s, 4H), 2.61 (s, 2H), 2.40 (s, 4H), 1.83 (s, 0H), 1.67 (s, ^1^H). ^13^C NMR (101 MHz, MeOD) δ 173.2, 172.3, 159.1, 142.4, 130.1 (d, J = 12.5 Hz), 129.2, 126.5, 118.2, 115.4, 52.0, 49.7, 39.4, 37.0, 33.1.

PAMAM-TPP-Cinnamic Acid (encapsulated): ^1^H NMR (400 MHz, MeOD) δ 7.50–7.40 (m, ^1^H), 7.32–7.20 (m, ^1^H), 6.40 (d, J = 16.0 Hz, ^1^H), 3.55–3.48 (m, ^1^H), 3.40 (t, J = 5.9 Hz, ^1^H), 3.01 (d, J = 6.1 Hz, ^1^H), 2.72 (s, ^1^H), 2.52 (s, ^1^H), 2.31 (d, J = 16.0 Hz, ^1^H), 1.72 (d, J = 7.8 Hz, ^1^H), 1.57 (s, ^1^H). ^13^C NMR (101 MHz, MeOD) δ 173.2, 171.4, 142.3, 135.1, 129.3, 128.5, 127.4, 121.5, 49.7, 36.9.

### 2.7. Physicochemical Characterization

Malvern Zetasizer Nano ZS90 (Malvern, UK) equipped with a He/Ne ion laser (l 633 nm) was employed to measure the particle sizes and zeta potentials of the PAMAM/Antioxidant complexes in ultrapure water solution. Lyophilized samples were dissolved in ultrapure water at a final concentration of 1 mg·mL^−1^. Solutions were gently vortexed and allowed to equilibrate for 30 min at room temperature. Prior to measurement, samples were filtered through 0.45 μm syringe filters to remove dust or large aggregates. Measurements were performed in disposable polystyrene cuvettes, and each sample was analyzed in triplicate, with PAMAM G_3_ as the control. In a typical laser-Doppler electrophoresis measurement, the average particle diameter was calculated by Stokes-Einstein equation from the particle velocity which was recorded by the laser light scattering technique; meanwhile, the zeta potential was calculated from the frequency shift induced electrophoretic mobility, which resulted from the Doppler effect. The frequency shift is defined as the shift of the scattered laser light relative to the original laser beam. Dynamic light scattering measurements were performed at 25 °C. The detection angle was fixed at 90–.

### 2.8. Radical Scavenging Assays

The antioxidant activity of PAMAM formulations was evaluated using the ABTS•+ and DPPH• radical-scavenging assays, which measure the capacity of antioxidants to scavenge free radicals [27,28]. The ABTS assay was performed following the procedure previously described [28], with modifications. A 7 mM ABTS stock solution was mixed with 2.45 mM potassium persulfate and allowed to react, protected from light for 16 h at room temperature to generate ABTS+ radical cation. Prior to analysis, the ABTS•+ solution was diluted with 80% methanol to obtain an absorbance of approximately 0.7 at 734 nm. Antioxidant solutions and PAMAM formulations, from a range of 0.01 to 0.5 mM, were mixed with the ABTS•+ reagent and incubated for 5 min in the dark. The decrease in absorbance at 734 nm was recorded using a UV-Vis spectrophotometer (Biobase Biodustry (Shandong) Co., Ltd., Jinan, China), and scavenging activity was calculated by comparing the absorbance of each sample with that of the control (ABTS•+ solution without antioxidant). The DPPH• assay was conducted according to the method previously described [27]. A 100 μM methanolic solution of DPPH• was diluted with 80% methanol to reach an absorbance of 0.8 at 517 nm. Various concentrations (0.01 to 0.5 mM) of each antioxidant and dendrimeric formulation were added to the DPPH• solution and incubated for 30 min in the dark. The reduction in absorbance at 517 nm, corresponding to the strong shift in color, was measured with the UV-Vis spectrophotometer. Antioxidant activity was expressed as the percentage decrease in absorbance relative to the control solution.

### 2.9. Molecular Dynamics of PAMAM-TPP-Antioxidant Compounds

Molecular dynamics simulations were performed to provide structural insight into dendrimer–phenolic acid interactions and to rationalize the physicochemical trends observed experimentally. PAMAM dendrimer groups, such as the ethylenediamine core and amidoamine monomers, were parameterized as reported previously using homemade scripts [29]. Three TPP groups were conjugated to three different and terminal groups. TPP groups, including alkyl moieties, were parameterized using VMD’s Force Field Toolkit (ffTK) version 1.3 [30]. This toolkit automates many aspects of the parametrization workflow while maintaining adherence to the rigorous methodological standards established by the CHARMM development community. Quantum mechanical calculations were performed using Gaussian 16 following the established CHARMM parametrization protocol [31]. The parametrization workflow consists of five sequential stages: initial topology generation, charge optimization, bonded parameter development, dihedral parameter fitting, and comprehensive validation. Target molecules were initially optimized at the B3LYP/6-31G(d) level of theory to obtain equilibrium geometries. The ffTK BuildPar module was employed to generate preliminary topology files by matching molecular fragments against the existing CHARMM parameter database. Novel atom types were identified through automated comparison with the CHARMM Chemical Component Dictionary (CGenFF), and missing parameters were flagged for subsequent development. Partial atomic charges were optimized to reproduce quantum mechanical water interaction energies, following the established CHARMM methodology. Water molecules were positioned at multiple orientations around polar and charged functional groups, with approximately 16–20 configurations generated per interaction site. Single-point interaction energies were calculated at the MP2/6-31G(d) level of theory for each water-solute configuration. Dihedral parameters were developed through systematic potential energy surface (PES) scanning. Each rotatable dihedral angle was scanned in 15° increments over a complete 360° rotation, yielding 24 data points per dihedral. Single-point energies were calculated at the MP2/6-31G(d)//B3LYP/6-31G(d) level of theory.

Each system was explicitly solvated in a periodic TIP3P water box with a minimum padding of 15 Å between the solute and the box boundaries. Sodium (Na^+^) and chloride (Cl^−^) ions were added to neutralize the net charge of each system and to establish a physiological ionic strength of 150 mM NaCl, using the Autoionize plugin of VMD. Three molecular systems were prepared, each containing 34 molecules of the corresponding antioxidant at a final concentration of approximately 30 mM. Each system underwent 5000 steps of energy minimization applying positional restraints of 10 kcal/mol·Å^2^ to the antioxidant molecules, which were progressively released over 1 ns of equilibration. A production run was then carried out at 310 K for 1000 ns. Temperature and pressure were maintained at 310 K and 1 atm, respectively, using the Nosé–Hoover thermostat and the Nosé–Hoover Langevin piston barostat with a semi-isotropic pressure coupling scheme (time constants: 0.1 ps for temperature, 1.0 ps for pressure). A 2 fs integration timestep was employed. Long-range electrostatic interactions were computed using the Particle Mesh Ewald (PME) method with a real-space cutoff of 1.2 nm, and a van der Waals cutoff of 1.2 nm with a smooth switching function beginning at 1.0 nm. All simulations were performed using NAMD 3.0 [30].

### 2.10. Cell Viability Studies

Cell viability assay was used to determine the cytotoxicity of PAMAM complexes. Cells from two different cell lines (HeLa and HEK293) were seeded in 96-well plates and cultured in Dulbecco’s Modified Eagle Medium (DMEM) supplemented with 10% (*v*/*v*) fetal bovine serum (FBS) and 1% (*v*/*v*) penicillin–streptomycin, under standard conditions (37 °C, humidified atmosphere with 5% CO_2_). The cells were incubated for 20–24 h until they reached 70–80% confluency, after which the growth media were removed and replaced with fresh media containing predetermined concentrations of PAMAM compounds (0.0001, 0.001, 0.01, 0.05, 0.1 and 1 mg/mL). Four hours later, the medium in each well was replaced with a fresh complete medium. After an additional 40–48 h of incubation, 25 µL of MTT stock solution (5 mg/mL in PBS) was added to each well. After a further 4 h of incubation with MTT, the medium was replaced with 150 µL of DMSO to dissolve the formazan crystal produced by live cells. The viable cells were counted by measuring the absorbance at 550 nm using an ELISA microplate reader (Bio-Rad Laboratories, Inc., Hercules, CA, USA). The cell viability was expressed as a percentage of the absorbance of the polymer-loading well to the polymer-free well.

### 2.11. In Vitro Cellular Assays of Dendrimer Antioxidant Capacity

Caffeic acid was selected for cellular antioxidant evaluation based on both experimental and computational criteria. Among the three tested compounds, caffeic acid exhibited the highest radical-scavenging activity in ABTS•+ and DPPH• assays, consistent with its greater degree of hydroxylation and intrinsic redox capacity. Molecular dynamics simulations further revealed that caffeic acid displayed the strongest association with the PAMAM–TPP dendrimer, characterized by the most favorable binding free energies, reduced solvent-accessible surface area, and increased interaction persistence relative to *p*-coumaric and cinnamic acids. These properties made caffeic acid the most suitable candidate to interrogate how dendrimer association modulates antioxidant accessibility and intracellular performance. Moreover, caffeic acid is well documented to exert cytoprotective and intracellular antioxidant effects, supporting its selection as a biologically relevant model compound for proof-of-concept cellular validation.

The antioxidant activity of different types of caffeic acid formulations was assessed in COS-7 cells, a well-established model for evaluating the cytotoxicity and cellular uptake of cationic polymer-based nanocarriers, including PAMAM dendrimers, and provide a reproducible baseline. These cells also exhibit high sensitivity to oxidative stress induced by H_2_O_2_ and are routinely used in DHE-based ROS quantification protocols, making them an appropriate model for evaluating the intracellular antioxidant activity of dendrimer formulations. Cells were seeded at 2 × 10^4^ cells/well in 96-well plates and allowed to adhere for 24 h. The test compounds (free caffeic acid, Caf/PAM(c), and Caf/PAM(e)) were added at a concentration of 10 µM and pre-incubated with cells for 2 h prior to oxidative challenge, to allow cellular uptake or surface association. After this pre-incubation period, cells were challenged with 600 µM H_2_O_2_ for 4 h in the continued presence of the test compounds. Total exposure time from first compound addition to ROS measurement was therefore 6 h. Cellular oxidative stress was evaluated immediately after the 4 h H_2_O_2_ incubation by DHE staining followed by epifluorescence microscopy. Controls included cells treated with each dendrimer alone (without H_2_O_2_), H_2_O_2_ alone, N-acetylcysteine (NAC, 1 mM) with H_2_O_2_, NAC alone, and untreated cells. All experimental conditions were evaluated using ten independent replicates (n = 10). It should be noted that this experimental design captures acute antioxidant performance under a single fixed time window; the kinetics of antioxidant release from dendrimer carriers may differ substantially over longer time periods, a point discussed further in Section 3.4.

### 2.12. Statistical Analysis

All experimental data are presented as mean ± standard error of the mean (SEM) unless otherwise stated. Statistical analyses were performed using Python 3.12 (SciPy library). Prior to hypothesis testing, data distributions were visually inspected to assess variability and outliers. For comparisons between two independent treatment groups under oxidative stress conditions, Welch’s two-tailed *t*-test was applied, as it does not assume equal variances between groups. Statistical significance was defined at α = 0.05. Exact *p*-values are reported where appropriate. Graphical representations and statistical summaries were generated using Matplotlib 3.10. No data points were excluded unless technically invalid, and all analyses were performed on raw background-corrected fluorescence values (MFI-BG).

## 3. Results and Discussion

### 3.1. Entrapment Efficiency and Physicochemical Properties

The encapsulation and covalent conjugation of phenolic antioxidants produced measurable changes in the physicochemical characteristics of the PAMAM-TPP formulations, as summarized in Table 1. DLS measurements are reported as intensity-weighted mean hydrodynamic diameters. It must be noted that the hydrodynamic diameters observed for all formulations (127–260 nm) are substantially larger than the expected size of individual G3 PAMAM dendrimers in aqueous solution. Based on literature values and the known dimensions of PAMAM dendritic architectures, a fully hydrated G3 PAMAM dendrimer with several shells of associated water is expected to present a hydrodynamic diameter of approximately 4–10 nm. The sizes measured here are therefore not consistent with isolated dendrimer molecules but rather indicate the presence of supramolecular aggregates comprising potentially tens to hundreds of individual dendrimer units. This is consistent with reports of PAMAM dendrimer association in aqueous media at moderate to high concentrations, driven by intermolecular hydrogen bonding, electrostatic interactions, and hydrophobic effects. The relatively high polydispersity index values (PdI 0.19–0.43) further support the presence of heterogeneous, multi-molecular assemblies rather than monodisperse individual macromolecules. DLS intensity distributions are biased toward larger scattering objects, which may additionally overestimate the apparent aggregate size.

Within supramolecular assemblies of this size, phenolic acid molecules could be located either (i) within the internal cavities of individual dendrimer units (classical encapsulation), (ii) electrostatically bound to the protonated surface amine groups (NH_3_^+^) of individual dendrimers within the aggregate, or (iii) partitioned between both environments. The acid–base properties of the system are relevant here: at the working pH (~7), the primary amine groups of PAMAM (pKa~9–10) are predominantly protonated (NH_3_^+^), while the carboxylic and phenolic groups of the antioxidants (pKa~4–10) may be partially or fully deprotonated. This creates a complementary electrostatic environment that could drive surface association in addition to, or instead of, internal encapsulation. We cannot exclude from the DLS and zeta potential data alone that a significant fraction of the associated antioxidant molecules is located at the dendrimer surface via electrostatic interactions. This possibility is supported by the persistence of positive zeta potential values across all formulations (+21 to +31 mV, Table 1), which indicates that despite antioxidant incorporation the surface charge was not neutralized, suggesting partial rather than complete coverage of surface amines.

Notwithstanding these limitations, several trends are observable within the dataset. Formulations involving covalent conjugation showed larger aggregate sizes on average (e.g., Caf/PAM(c): 260 ± 41 nm) compared to their encapsulated counterparts (Caf/PAM(e): 239 ± 37 nm), possibly reflecting surface modification-induced changes in intermolecular association behavior. However, given the large standard deviations and the aggregate nature of the particles, fine-grained comparisons between individual formulations should be interpreted cautiously. Notably, zeta potential values for covalent conjugates showed a slight decrease relative to encapsulated systems, potentially consistent with partial neutralization of surface amines by covalently attached carboxylate-bearing antioxidants, in line with previous reports [32,33]. Moderate surface charge values indicate electrostatic colloidal stabilization, supported by PdI values below 0.4 for most formulations [34]. Encapsulation efficiency, quantified by HPLC from the unentrapped antioxidant fraction after ultrafiltration, provides a complementary measure of antioxidant association that is independent of DLS size interpretation. Caffeic acid, p-coumaric acid, and cinnamic acid exhibited association efficiencies of 93.8%, 78.9%, and 71%, respectively, indicating that the majority of each compound became associated with the PAMAM-TPP material after the encapsulation procedure. These values reflect the total quantity of antioxidant retained by the dendrimer assemblies (both surface-associated and internally encapsulated fractions), and their trend is consistent with the molecular dynamics predictions of relative binding affinity discussed in Section 3.2. The higher association efficiency of caffeic acid may reflect its greater number of hydrogen-bond donor groups and stronger overall electrostatic and non-covalent interactions with both the interior and the surface of the dendrimer. The relatively similar association efficiencies of p-coumaric acid (78.9%) and cinnamic acid (71%) suggest that factors beyond phenolic hydroxyl group count—such as π–π stacking interactions with aromatic residues or hydrophobic effects—also contribute to retention within the dendrimer assembly, a point further addressed in Section 3.2.

### 3.2. Molecular Dynamics Simulations

**Figure 3:**
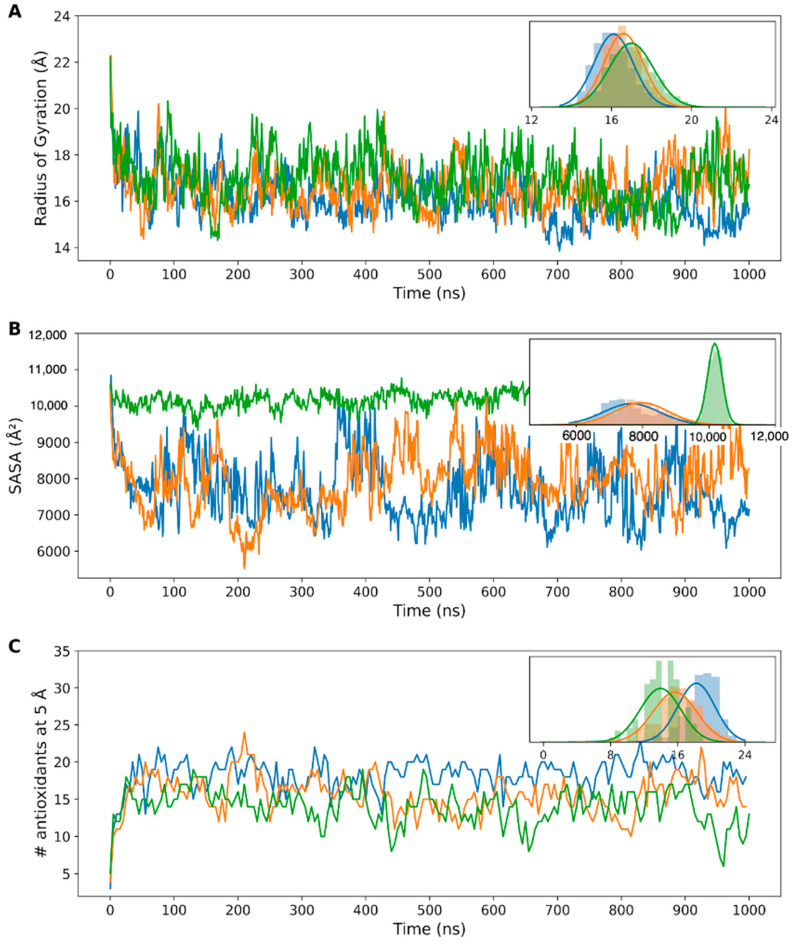
(**A**) Radius of gyration of the dendrimer in the system with caffeic acid molecules (blue), *p*-coumaric acid molecules (orange), and cinnamic acid molecules (green), measured over the trajectory. (**B**) Average solvent-accessible surface area (SASA) of all antioxidant molecules in each system (blue = caffeic, orange = *p*-coumaric, green = cinnamic), throughout the simulation. (**C**) Number (#) of antioxidant molecules within a 5 Å radius of the dendrimer in each system (blue = caffeic, orange = *p*-coumaric, green = cinnamic), measured over the course of the molecular dynamics simulation. Insets show the distribution of the data for each plot.

Based on the molecular dynamics simulations carried out over 1 μs for each encapsulated antioxidant/PAMAM system (Caf/PAM(e), Cin/PAM(e), Cou/PAM(e)), parameters associated with the degree of dendrimer compaction in the presence of the different antioxidants were obtained. As shown in Figure 3A, in the presence of cinnamic acid—the most hydrophobic antioxidant—the average radius of gyration was slightly higher than for dendrimers in the other systems, indicating lower compaction. This suggests decreased stability of the dendrimer–antioxidant complex in this case. Furthermore, the average SASA values revealed that in the system with cinnamic acid, molecules remained more solvent-accessible throughout the simulation, suggesting poor encapsulation by the PAMAM-TPP dendrimer. This is likely due to the low hydrophilicity of cinnamic acid, which contrasts with the dendrimer’s polar, amino-rich terminal groups. In contrast, caffeic and *p*-coumaric acids exhibited lower SASA values and smaller dendrimer radii of gyration, suggesting better encapsulation and more stable interactions. This is consistent with their higher polarity and better complementarity with the dendrimer’s chemical nature.

Regarding the number of interacting molecules, data in Figure 3C (and inset) showed that approximately 20 caffeic acid molecules consistently interacted with the dendrimer, compared to 16 *p*-coumaric acid and 13 cinnamic acid molecules. The combination of lower interaction numbers and high SASA in the cinnamic acid system indicates transient or superficial interactions, where antioxidants may escape from the dendrimer and be replaced over time.

**Figure 4:**
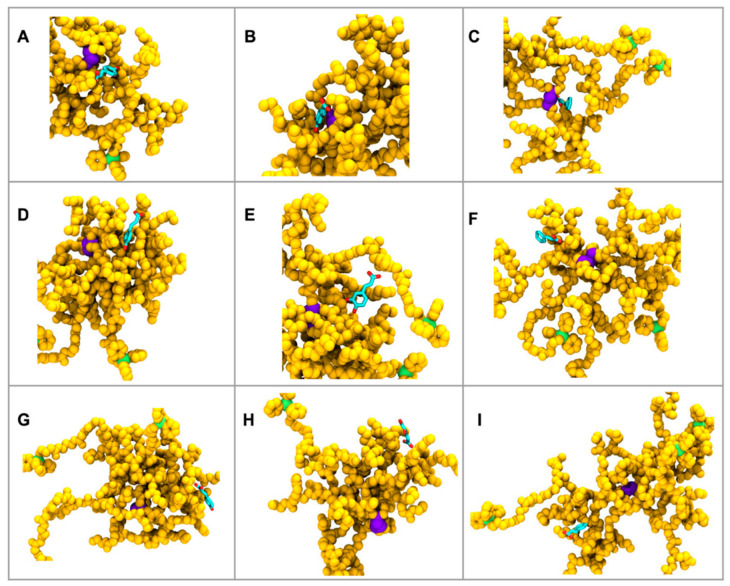
Snapshots from molecular dynamics simulations displaying the spatial distribution of antioxidant molecules relative to the PAMAM-TPP dendrimer core. Columns (**A**,**D**,**G**): caffeic acid. Columns (**B**,**E**,**H**): p-coumaric acid. Columns (**C**,**F**,**I**): cinnamic acid. Rows represent antioxidants located at short distance ((**A**–**C**), ~4–6 Å from core)), intermediate distance ((**D**–**F**), ~11–13 Å)), and long distance ((**G**–**I**), ~18–22 Å)) from the dendrimer core. The dendrimer core is shown in purple, while the chains are in yellow; TPP groups in green; antioxidant molecules in cyan (carbon) and red (oxygen).

Binding free energy calculations (Table 2) supported these findings. Near the dendrimer core, all molecules showed strong binding, with caffeic acid demonstrating the highest affinity due to more favorable van der Waals interactions. At intermediate distances—representing more hydrophobic zones—caffeic acid’s affinity dropped markedly, while cinnamic acid showed improved binding, consistent with its lipophilic nature (as shown in Figure 4). At peripheral distances, all antioxidants exhibited moderate affinity, being partially shielded by the dendrimer’s outer groups but more exposed to solvent, reflecting intermediate binding behavior. While cinnamic acid lacks phenolic hydroxyl groups and showed lower overall interaction counts and higher SASA values than caffeic acid, its MM-GBSA binding free energy at intermediate distance from the core (~12 Å, ΔG = −22.9 kcal/mol) is more favorable than that of caffeic acid (−21.2 kcal/mol) and markedly more favorable than p-coumaric acid (−8.2 kcal/mol) at the equivalent region. This observation reflects the distance-dependent chemical composition of the PAMAM dendrimer interior. The intermediate zone (~10–15 Å from core) corresponds to a region rich in hydrophobic methylene groups and amide bonds with limited solvent accessibility, which is chemically complementary to the more lipophilic cinnamic acid scaffold. In this zone, van der Waals interactions—as reflected in the ΔEVdW component (−7.1 kcal/mol for cinnamic acid at ~12 Å)—dominate over electrostatic contributions. In contrast, the highly polar caffeic acid benefits from stronger electrostatic interactions closer to the water-exposed dendrimer surface. These region-specific binding preferences underscore that antioxidant–dendrimer affinity is not determined solely by the number of hydroxyl groups but also by the spatial complementarity between the antioxidant’s physicochemical properties and the local chemical environment at different depths within the dendrimer architecture. This is also relevant for interpreting the encapsulation efficiency data: the relatively moderate but non-negligible association of cinnamic acid (71%) likely reflects contributions from hydrophobic partitioning and π–π stacking in addition to hydrogen-bonding interactions.

In summary, molecular dynamics simulations and binding energy analysis suggest that PAMAM-TPP dendrimers interact more effectively with polar antioxidants such as caffeic and *p*-coumaric acids. Caffeic acid, in particular, shows a strong tendency to locate in the inner regions of the dendrimer, consistent with its higher binding affinity and stronger encapsulation behavior.

These computational predictions align well with experimental encapsulation efficiencies. Caffeic acid, which showed the highest number of stable interactions (~20 molecules) and lowest SASA values in MD simulations, exhibited an entrapment efficiency of 93.8%. In contrast, cinnamic acid, with fewer dendrimer interactions (~13 molecules) and higher solvent exposure, showed significantly lower encapsulation efficiency (71%). This experimental–computational concordance validates the predictive capacity of our MD approach and confirms that binding affinity directly correlates with encapsulation success.

### 3.3. Antioxidant Activity

**Figure 5:**
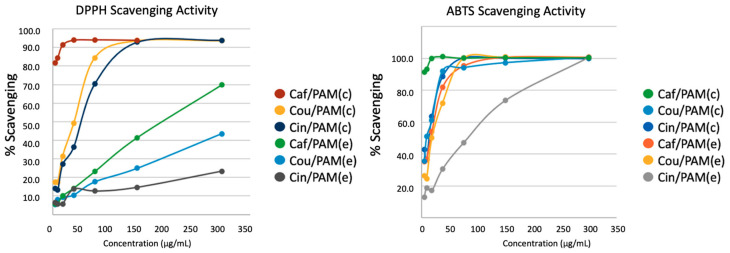
In vitro radical scavenging activity of caffeic acid and PAMAM-based formulations evaluated by DPPH• (left panel) and ABTS•+ (right panel) assays. Free caffeic acid (Caf), covalent PAMAM–TPP–caffeic acid conjugate (Caf/PAM(c)), and non-covalent encapsulated system (Caf/PAM(e)) were tested over a range of concentrations. Radical scavenging activity is expressed as percentage inhibition relative to the corresponding radical control. Data represent mean values of independent measurements.

The antioxidant performance of PAMAM–TPP dendrimer formulations was evaluated using both in vitro radical-scavenging assays (DPPH• and ABTS•+) and a cellular oxidative stress model, thereby enabling comparison of intrinsic chemical reactivity with intracellular functional efficacy. Consistent with the phenolic structures of the free compounds, radical-scavenging activity followed the trend caffeic > *p*-coumaric > cinnamic acid, consistent with the increasing number of hydroxyl groups and their resonance-stabilization capacity, which has been widely documented to enhance radical scavenging [35,36,37]. Incorporation into PAMAM dendrimers did not substantially alter the maximal radical scavenging capacity of the antioxidants, as both encapsulated and covalently bound systems achieved near-complete DPPH• and ABTS•+ neutralization at the highest tested concentrations (Figure 5) [38]. However, differences in scavenging kinetics were observed, with encapsulated systems displaying a slight delay in initial radical quenching, likely due to diffusion-limited accessibility of phenolic groups, whereas covalent conjugation promoted more sustained radical trapping over time. These differences suggest that the mode of association modulates the temporal availability and spatial accessibility of the antioxidant moieties rather than their intrinsic chemical reactivity.

DPPH• scavenging assays revealed differences in antioxidant performance among encapsulated formulations. Caf/PAM(e) exhibited the highest radical-scavenging capacity, achieving ~90–95% DPPH• inhibition at 350 µg/mL, followed by Cou/PAM(e) (~85–90%), while Cin/PAM(e) showed lower activity (~70%). For caffeic and p-coumaric acid formulations, this trend is consistent with the contribution of phenolic hydroxyl groups to radical scavenging: caffeic acid (two –OH groups) outperforms p-coumaric acid (one –OH group) as expected from their hydrogen-atom transfer capacity [35,36,37]. Although cinnamic acid lacks phenolic hydroxyl groups, it still shows moderate DPPH• inhibition (~70%), and its Table 2 binding free energies at intermediate dendrimer distances (ΔG = −22.9 kcal/mol) are comparable to or exceed those of caffeic acid in the same region, driven predominantly by van der Waals interactions with the hydrophobic dendrimer interior (see Section 3.2). The residual radical-scavenging activity of Cin/PAM(e) may therefore reflect a combination of partial PAMAM carrier activity—as PAMAM dendrimers have been reported to contribute to radical quenching in certain systems [39]—and partial solvent exposure of cinnamic acid molecules transiently located at the dendrimer periphery. These data indicate that while the number of phenolic hydroxyl groups is the primary driver of intrinsic radical-scavenging capacity for free compounds, the supramolecular organization within dendrimer assemblies introduces additional variables—including molecular accessibility, binding localization, and carrier contributions—that modulate the observed activity in a manner that does not follow a simple structure–activity relationship based on hydroxylation degree alone.

### 3.4. Cellular Studies

To assess whether these effects translated into cellular antioxidant efficacy, caffeic acid and its PAMAM-based formulations were selected for in vitro testing in COS-7 cells subjected to hydrogen peroxide-induced oxidative stress. Two dendrimeric systems were evaluated: Caf/PAM(c), a covalent conjugate, and Caf/PAM(e), a non-covalent encapsulated formulation. Free caffeic acid, NAC + H_2_O_2_, and untreated cells were included as controls.

**Figure 6:**
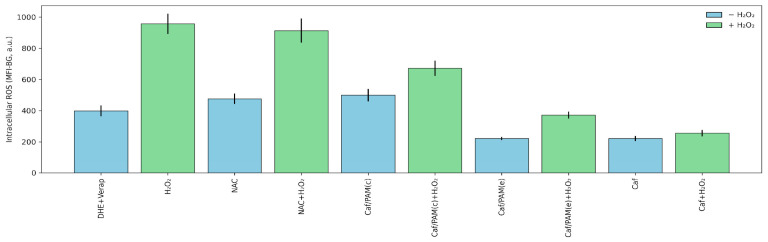
Intracellular reactive oxygen species (ROS) levels in COS-7 cells subjected to oxidative stress and treated with caffeic acid and PAMAM-based formulations. Cells were exposed to 600 µM H_2_O_2_ for 4 h in the presence of free caffeic acid (Caf), covalent PAMAM-TPP–caffeic acid conjugates (Caf/PAM(c)), or non-covalent encapsulated systems (Caf/PAM(e)). Intracellular ROS levels were quantified by dihydroethidium (DHE) fluorescence and analyzed by epifluorescence microscopy. Control conditions included untreated cells, H_2_O_2_ alone, NAC, and NAC + H_2_O_2_. Data are presented as mean ± SEM of background-corrected fluorescence intensity (MFI-BG, arbitrary units). Statistical comparisons between antioxidant treatments under oxidative stress were performed using Welch’s two-tailed *t*-test, with significance assessed at α = 0.05.

Quantitative analysis of intracellular ROS levels, measured by Dihydroethidium (DHE) fluorescence corrected for background (MFI-BG), revealed clear differences among treatments under oxidative stress conditions (Figure 6). While all antioxidant treatments significantly reduced ROS levels relative to H_2_O_2_ alone, free caffeic acid exhibited the lowest MFI-BG values, indicating the most effective immediate ROS neutralization. In contrast, both PAMAM-associated formulations displayed higher ROS levels than free caffeic acid. Welch’s *t*-test analysis confirmed that Caf/PAM(c) showed significantly higher MFI-BG values than free caffeic acid (t = −7.83, *p* = 7.38 × 10^−10^), and that Caf/PAM(e) was also significantly less effective than free caffeic acid (t = −3.85, *p* = 1.82 × 10^−4^). Direct comparison between dendrimeric formulations further revealed that Caf/PAM(e) outperformed the covalent conjugate PAMAM–TPP–Caf(c) (t = 5.61, *p* = 1.19 × 10^−6^), although neither matched the antioxidant efficacy of free caffeic acid under acute oxidative stress.

When interpreted alongside molecular dynamics simulations, these findings reveal a mechanistic trade-off between antioxidant retention and functional bioavailability. MD analyses showed that caffeic acid exhibits strong association with the PAMAM–TPP dendrimer, characterized by reduced solvent accessibility, increased contact persistence, and preferential localization within the dendrimer interior. While such encapsulation enhances stability and retention, it is expected to limit the immediate accessibility of phenolic groups to reactive oxygen species and to slow antioxidant release during short experimental timeframes. This suggests a mechanistic explanation for the superior performance of free caffeic acid in acute H_2_O_2_ challenge assays, despite its weaker retention relative to dendrimer-associated forms.

In the case of covalent conjugates, although the antioxidant moieties are positioned closer to the dendrimer periphery, covalent attachment imposes conformational constraints and may restrict optimal orientation or flexibility of phenolic groups toward ROS. This steric and dynamic restriction likely contributes to the reduced acute activity of Caf/PAM(c) relative to the encapsulated formulation. Importantly, the fact that covalently conjugated systems retain measurable antioxidant activity indicates that phenolic groups are not fully shielded upon attachment but remain at least partially solvent-accessible. This interpretation is consistent with the MD-derived solvent accessibility values and supports the notion that dendrimer conjugation modulates, rather than abolishes, antioxidant functionality.

Importantly, none of the PAMAM-based formulations increased ROS levels in the absence of H_2_O_2_, confirming their biocompatibility and indicating that the observed effects reflect genuine antioxidant behavior rather than cytotoxicity or assay-related fluctuations. Overall, these results suggest that PAMAM–TPP dendrimers effectively retain and stabilize caffeic acid but attenuate its immediate intracellular ROS-scavenging capacity, highlighting the importance of balancing antioxidant retention with release kinetics when designing dendrimer-based delivery systems. Taken together, these observations support a model in which conjugation and encapsulation impose distinct structural and dynamic constraints on antioxidant molecules, ultimately governing their temporal availability and biological performance.

### 3.5. Citotoxicity Assay

Cell viability studies (Supplementary Figure S2) demonstrated that all PAMAM-TPP formulations maintained >80% viability at concentrations up to 0.1 mg/mL in both HeLa and HEK293 cell lines. A decrease related to concentration was observed at 1 mg/mL, consistent with previous reports describing dose-dependent cytotoxicity of cationic PAMAM dendrimers [40]. Importantly, the concentrations used for antioxidant and ROS experiments were below cytotoxic thresholds, supporting the fact that the observed intracellular effects were not due to nonspecific toxicity but rather to genuine modulation of oxidative stress.

## 4. Conclusions

This study demonstrates that third-generation PAMAM–TPP dendrimers are versatile nanocarriers capable of both encapsulating and covalently binding phenolic antioxidants, with molecular dynamics simulations providing detailed insight into the structural and energetic determinants of antioxidant–dendrimer interactions. Among the antioxidants studied, caffeic acid exhibited the strongest binding affinity, highest encapsulation efficiency, and most stable association with the dendrimer, consistent with its higher polarity and capacity for hydrogen bonding.

Importantly, integration of computational, chemical, and cellular data revealed a functional trade-off between antioxidant retention and immediate intracellular efficacy. While PAMAM–TPP association enhances antioxidant stabilization and sequestration, it reduces short-timescale ROS neutralization under acute oxidative stress conditions, as free caffeic acid consistently outperformed dendrimer-associated formulations in cellular assays. Encapsulated systems exhibited intermediate behavior, whereas covalent conjugation imposed the greatest restriction on antioxidant accessibility, highlighting the critical influence of conjugation mode on biological outcome.

These findings underscore that strong encapsulation and binding affinity do not necessarily translate into superior acute antioxidant performance, and that the temporal and spatial availability of antioxidants must be considered alongside stability when designing nanocarrier systems. PAMAM–TPP dendrimers therefore represent a promising platform for applications where controlled retention, protection from premature degradation, or sustained release are desired, rather than immediate ROS scavenging. Overall, this work provides a mechanistic framework linking dendrimer–antioxidant interactions to functional cellular outcomes and emphasizes the importance of rational nanocarrier design to balance stability, release kinetics, and biological efficacy in oxidative stress-related applications.

## Figures and Tables

**Table 1:** Dynamic Light-Scattering (DLS) measurements for average size, zeta potential and polydispersity index for all prepared compounds.

Compound	Average Size (nm)	Zeta Potential (mV)	PdI
PAMAM-TPP	183 ± 8	23.0 ± 0.9	0.35 ± 0.01
Caf/PAM(c)	260 ± 41	26.0 ± 0.3	0.26 ± 0.05
Cou/PAM(c)	168 ± 8	24.0 ± 0.6	0.43 ± 0.12
Cin/PAM(c)	178 ± 57	26.5 ± 0.6	0.32 ± 0.05
Caf/PAM(e)	239 ± 37	30.9 ± 3.3	0.25 ± 0.03
Cou/PAM(e)	127 ± 37	22.2 ± 0.9	0.19 ± 0.01
Cin/PAM(e)	178 ± 57	21.1 ± 2.0	0.35 ± 0.14

**Table 2:** MM-GBSA Binding free energy and their components (electrostatic + solvation and Van der Waals) obtained for dendrimers and antioxidants at different distances from the core of the dendrimer.

Antioxidant	Distance to Core (Å)	ΔE_electrostatic_ + ΔG_solv_ (kcal/mol)	ΔE_VdW_ (kcal/mol)	MM-GBSA ΔG_binding_ (kcal/mol)
Caffeic	~4	−9.3	−8.5	−17.7 ± 1.0
	~11	−7.8	−13.4	−21.2 ± 0.9
	~18	−10.5	−2.9	−13.4 ± 0.7
*p*-coumaric	~5	−8.5	−14.0	−22.5 ± 1.5
	~13	4.5	−12.7	−8.2 ± 0.7
	~22	−6.0	−5.8	−11.9 ± 1.1
Cinnamic	~6	−6.8	−9.0	−15.7 ± 0.9
	~12	−15.8	−7.1	−22.9 ± 1.2
	~18	−7.9	−8.2	−16.1 ± 2.2

## Data Availability

Data presented in this study are available in the article and Supplementary Materials.

## Supplementary Materials

The following supporting information can be downloaded at: https://www.mdpi.com/article/10.3390/polym18091086/s1, Figure S1: 1H-NMR spectra for the different prepared complexes. (a–c) corresponds to antioxidant (caffeic, coumaric and cinnamic acid, respectively) bound to PAMAM surface through EDC/NHS method. (d–f) are for antioxidants (caffeic, coumaric and cinnamic acid, respectively) and TPP, all bound through EDC/NHS method, while (g–i) are the prepared complexes with chemically bound TPP and encapsulated antioxidants (caffeic, coumaric and cinnamic acid, respectively) through electrostatic interactions; Figure S2: Cell viability at 24 h of prepared compounds using HEK and HeLa cell lines.
