# Supplementary material for: Experimental and Computational Analysis of Phenolic Acid Association with PAMAM Dendrimers: Comparing Different Formulation Techniques
Source: Polymers (Basel). 2026 Apr 29;18(9):1086. doi: 10.3390/polym18091086 (PMC13165792; doi:10.3390/polym18091086)
Supplement: Supplementary file 1 [file polymers-18-01086-s001.zip › polymers-4250583-supplementary.pdf]

Supplementary Material.

# Experimental and Computational Analysis of Phenolic Acid Association with PAMAM Dendrimers: Comparing Different Formulation Techniques

**Christopher Sbarbaro [1], Ma. Andreina Rangel-Ramírez [2], Emilio Salas [1], Francisco Salgado [1], María Carolina Otero [3], Alvaro A. Elorza [4], Fernando González-Nilo [1], Valeria Márquez-Miranda [1,*] and Yorley Duarte [1,5,*]**

[1] Center of Bioinformatics and Integrative Biology, Facultad de Ciencias de la Vida, Universidad Andres Bello, Santiago 8370035, Chile; christopher.sbarbaro@usach.cl (C.S.); e.salasaguilar@uandresbello.edu (E.S.); fsalgado@uchile.cl (F.S.); fernando.gonzalez@unab.cl (F.G.-N.)
[2] Departamento de Ciencias Biológicas, Facultad de Ciencias de la Vida, Universidad Andrés Bello,
Santiago 8370035, Chile; rangelmariandre@gmail.com
[3] Escuela de Química y Farmacia, Facultad de Medicina, Universidad Andrés Bello, Santiago 8370035, Chile; maria.otero@unab.cl
[4] Instituto de Ciencias Biomédicas, Facultad de Medicina y Ciencias de la Vida, Universidad Andrés Bello, Santiago 8370035, Chile; alvaro.elorza@unab.cl
[5] Instituto de Neurociencias, Centro Interdisciplinario de Neurociencias de Valparaíso, Universidad de
Valparaíso, Valparaíso 2360102, Chile
* Correspondence: valeria.marquez@unab.cl (V.M.-M.); yorley.duarte@unab.cl (Y.D.)

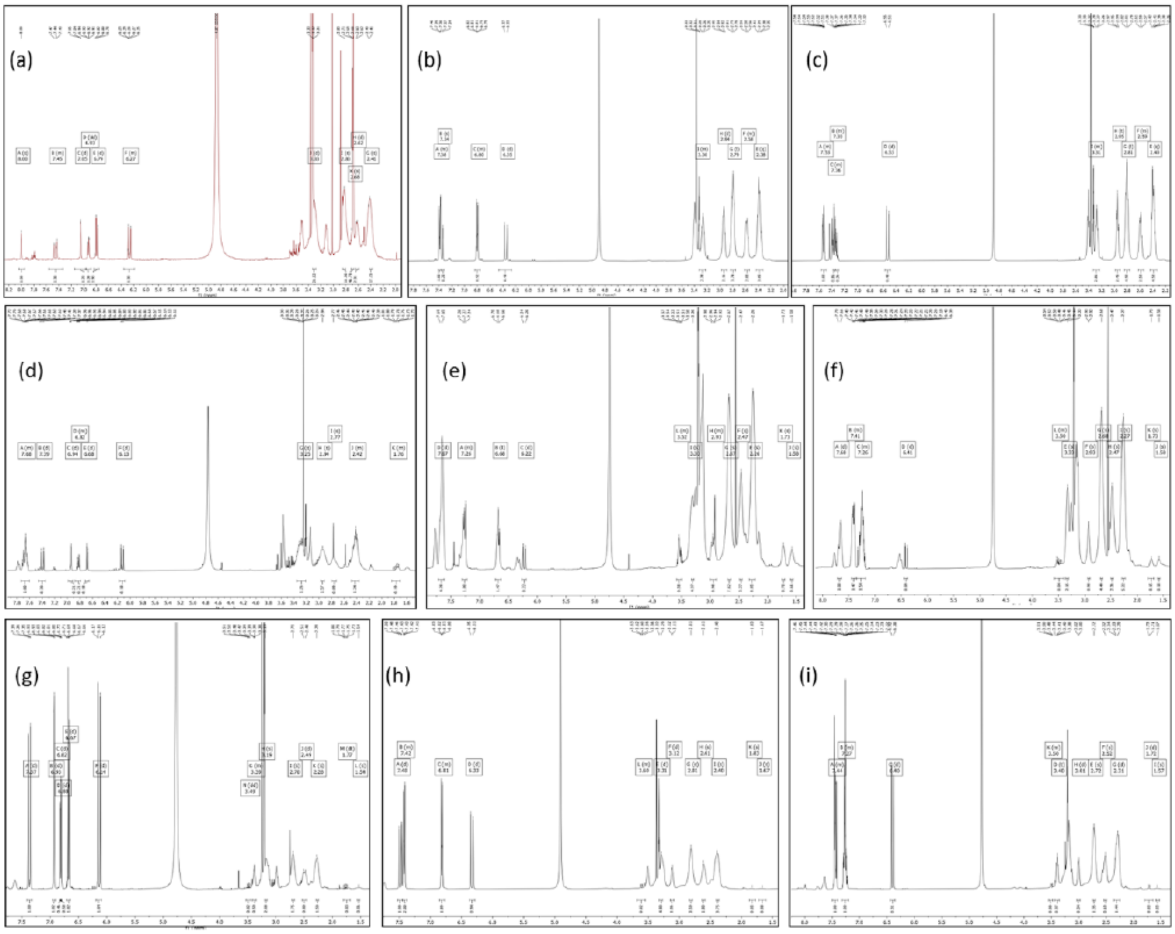


Supplementary Figure S1: 1H-NMR spectra for the different prepared complexes. a-c corresponds to antioxidant (caffeic, coumaric and cinnamic acid, respectively) bound to PAMAM surface through EDC/NHS method. d-f are for antioxidants (caffeic, coumaric and cinnamic acid, respectively) and TPP, all bound through EDC/NHS method, while g-i are the prepared complexes with chemically bound TPP and encapsulated antioxidants (caffeic,coumaric and cinnamic acid, respectively) through electrostatic interactions.

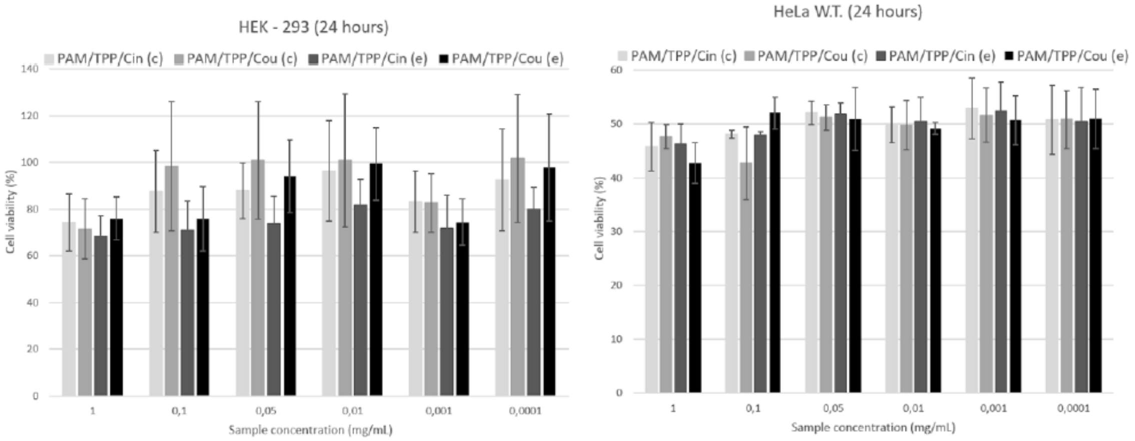


Supplementary Figure S2: Cell viability at 24 hours of prepared compounds using HEK and HeLa cell lines.
